# New operational taxonomic units of *Enterocytozoon* in three marsupial species

**DOI:** 10.1186/s13071-018-2954-x

**Published:** 2018-06-28

**Authors:** Yan Zhang, Anson V. Koehler, Tao Wang, Shane R. Haydon, Robin B. Gasser

**Affiliations:** 1 Department of Veterinary Biosciences, Melbourne Veterinary School, Faculty of Veterinary and Agricultural Sciences, The University of Melbourne, Parkville, Victoria 3010 Australia; 2 Melbourne Water, Docklands, Victoria 3001 Australia

**Keywords:** *Enterocytozoon bieneusi*, Operational taxonomic units, Genotypes, Prevalence, Eastern grey kangaroo, Swamp wallaby, Common wombat, Australia

## Abstract

**Background:**

*Enterocytozoon bieneusi* is a microsporidian, commonly found in animals, including humans, in various countries. However, there is scant information about this microorganism in Australasia. In the present study, we conducted the first molecular epidemiological investigation of *E. bieneusi* in three species of marsupials (*Macropus giganteus*, *Vombatus ursinus* and *Wallabia bicolor*) living in the catchment regions which supply the city of Melbourne with drinking water.

**Methods:**

Genomic DNAs were extracted from 1365 individual faecal deposits from these marsupials, including common wombat (*n* = 315), eastern grey kangaroo (*n* = 647) and swamp wallaby (*n* = 403) from 11 catchment areas, and then individually tested using a nested PCR-based sequencing approach employing the internal transcribed spacer (*ITS*) and small subunit (*SSU*) of nuclear ribosomal DNA as genetic markers.

**Results:**

*Enterocytozoon bieneusi* was detected in 19 of the 1365 faecal samples (1.39%) from wombat (*n* = 1), kangaroos (*n* = 13) and wallabies (*n* = 5). The analysis of *ITS* sequence data revealed a known (designated NCF2) and four new (MWC_m1 to MWC_m4) genotypes of *E. bieneusi*. Phylogenetic analysis of *ITS* sequence data sets showed that MWC_m1 (from wombat) clustered with NCF2, whereas genotypes MWC_m2 (kangaroo and wallaby), MWC_m3 (wallaby) and MWC_m4 (kangaroo) formed a new, divergent clade. Phylogenetic analysis of *SSU* sequence data revealed that genotypes MWC_m3 and MWC_m4 formed a clade that was distinct from *E. bieneusi.* The genetic distinctiveness of these two genotypes suggests that they represent a new species of *Enterocytozoon*.

**Conclusions:**

Further investigations of *Enterocytozoon* spp*.* from macropods and other animals will assist in clarifying the taxonomy and epidemiology of these species in Australia and elsewhere, and in assessing the public health risk of enterocytozoonosis.

**Electronic supplementary material:**

The online version of this article (10.1186/s13071-018-2954-x) contains supplementary material, which is available to authorized users.

## Background

*Enterocytozoon bieneusi* (Microsporidia) is a tiny (0.5 × 1.5 μm) [1] fungus or fungus-like microorganism [2, 3]. This microorganism infects the gastrointestinal tracts of mammals, and can cause acute or chronic diarrhoea, wasting and/or malabsorption [4–7]. However, some individuals infected with *E. bieneusi* do not exhibit clinical signs, and can thus represent carriers with the potential of disseminating *E. bieneusi* spores (*via* faeces) into the environment and/or to other people or animals [8]. As an opportunistic pathogen, *E. bieneusi* infects both immunocompromised (e.g. HIV/AIDS and organ transplant patients) and immunocompetent people [1], and has been recorded in at least 74 species of animals (e.g. artiodactyls, carnivores, lagomorphs, perissodactyls, primates and rodents) [9].

The accurate classification of *E. bieneusi* from biological matrices (faeces, soil and water) is not possible using phenetic (e.g. morphological) approaches, such that molecular tools need to be employed. Specifically, PCR-based sequencing of the internal transcribed spacer (*ITS*) of nuclear ribosomal DNA is an established, widely used method for the specific and genotypic identification and taxonomic classification of *E. bieneusi* [1]. Using this tool, more than 200 genotypes of *E. bieneusi* have been defined, 52 of which have been recorded only in humans, 34 in both humans and animals, 103 exclusively in animals (including artiodactyls, carnivores, lagomorphs, perissodactyls, primates and rodents) and 36 in water samples [9]. To date, there are only two studies reporting *E. bieneusi* in marsupials, but both studies involved captive kangaroos in China [10, 11].

Some genotypes, including D, EbpA, J, K and Type IV, have been identified in both humans and animals (e.g. bovids, canids and primates), and are thus recognised as potentially zoonotic [1, 12]. However, there has been no experimentation to demonstrate cross-species transmissibility [13]. Nonetheless, particular attention needs to be paid to these ‘zoonotic’ genotypes in the context of food- or water-borne disease (enterocytozoonosis) [14, 15]. Indeed, *E. bieneusi* is recognised as a Category B Priority Pathogen by National Institute of Allergy and Infectious Diseases (NIAID) and a ‘water contaminant candidate’ by the United States Environmental Protection Agency [6]. However, *E. bieneusi* is not mentioned in the Australian Drinking Water Guidelines (www.nhmrc.gov.au/guidelines-publications/eh52; accessed 28 February 2018), and there is no detailed information on its relevance as a waterborne pathogen for most countries.

Melbourne (population: ~4 million people) in the state of Victoria, Australia, is one of the few cities in the world that receives largely unfiltered drinking water from protected wilderness catchment areas. The management of these areas includes long water retention times, a monitoring programme for pathogens in source water and restricted access for humans. These catchment areas contain native and feral animals, and the monitoring of zoonotic microbes is central to the prevention of waterborne disease or disease outbreaks.

Since 1997, we have been investigating selected waterborne microbes, including *Cryptosporidium* and *Giardia*, in faecal samples from animals inhabiting these catchment areas [16]. As there was no published information on *E. bieneusi* in Australia, except for case studies of human enterocytozoonosis in HIV/AIDS patients (e.g. [17, 18]), we extended our monitoring programme to include this microbe. Recently, we conducted the first molecular epidemiological survey of *E. bieneusi* in wild fallow, red and sambar deer [19], which are abundant animals within Melbourne’s drinking water catchment areas (cf. [16, 20]). *Enterocytozoon bieneusi* was detected in 25 of the 610 (4.1%) samples tested, and was found exclusively in sambar deer. Other animals, such as marsupials, rabbits, canids and birds, are also relatively pronounced in these catchments, and might represent potential carriers of *E. bieneusi*. For this reason, in this study, we explored the prevalence, genetic diversity and zoonotic potential of *Enterocytozoon* in wombats, kangaroos and wallabies using a nested PCR-based sequencing of *ITS* and small subunit of nuclear ribosomal DNA gene (*SSU*), and phylogenetic analysis of sequence data sets. We discuss the research findings in the context of *Enterocytozoon* taxonomy and epidemiology.

## Methods

### Melbourne’s water catchments

Natural water catchments, covering more than 160,000 hectares, supply drinking water to ~4 million people in the city of Melbourne and environs. Humans and domestic animals have restricted access to most catchment areas. Water is treated in accord with national and international guidelines (Australian Drinking Water Guidelines www.nhmrc.gov.au/guidelines-publications/eh52; accessed February 2018). The catchment areas studied here were Armstrong (AM) 37°38'S, 145°51'E; Cardinia (CA) 37°47'S, 145°24'E; Greenvale (GV) 37°37'S, 144°54'E; Maroondah (MR) 37°38'S, 145°33'E; O’Shannassy (OS) 37°40'S, 145°48'E; Silvan (SV) 37°50'S, 145°25'E; Sugarloaf (SL) 37°40'S, 145°18'E; Tarago (TAR) 37°59'S, 145°55'E; Thompson (TH) 37°47'S, 146°21'E; Upper Yarra (UY) 37°40'S, 145°55'E and Yan Yean (YY) 37°33'S, 145°08'E (cf. [16]. CA, GV and SV act as storage facilities for the larger catchments; MR, OS, TH and UY are located in the densely forested Yarra Ranges catchment; TAR is an ‘open’ water-supply catchment abutting farmland; and YY is a much smaller catchment surrounded by residential areas.

### Samples and DNA isolation

A total of 1365 faecal deposits from marsupials, including common wombat (*Vombatus ursinus*; *n* = 315), eastern grey kangaroo (*Macropus giganteus*; *n* = 647) and swamp wallaby (*Wallabia bicolor*; *n* = 403) were collected from eleven water catchments from September 2009 to March 2017 (Additional file 1: Table S1). Genomic DNA was isolated directly from 0.25 g of each of the 1365 samples using the PowerSoil kit (MoBio, Carlsbad, CA, USA) according to the manufacturer’s instructions. The host origin of scats was identified using a field guide [21], and verified by testing faecal DNA employing a nested PCR-based sequencing approach using the mitochondrial cytochrome *b* gene (cf. [22]).

### Nested PCR-based sequencing of *E. bieneusi ITS*

From individual faecal DNA samples, the internal transcribed spacer (*ITS*) of nuclear ribosomal DNA of *E. bieneusi* was specifically amplified using degenerate primers originally published by Katzwinkel-Wladarsch et al. [23]. In the first round, primers MSP-1 (forward: 5'-TGA ATG KGT CCC TGT-3') and MSP-2B (reverse: 5'-GTT CAT TCG CAC TAC T-3') were employed to amplify 601 bp of *ITS* plus flanking gene sequences. In the second round, primers MSP-3 (forward: 5'-GGA ATT CAC ACC GCC CGT CRY TAT-3') and MSP-4B (reverse: 5'-CCA AGC TTA TGC TTA AGT CCA GGG AG-3') were used to amplify a product of ~535 bp containing 130 bp of the 3'-end of the small subunit (*SSU*) of the nuclear rDNA gene, ~243 bp of the internal transcribed spacer (*ITS*) plus 162 bp of the 5'-region of the large subunit (*LSU*) rDNA gene.

Nested PCR for amplification of *ITS* was conducted in 50 μl in a standard buffer containing 2.0 μM MgCl_2,_ 0.4 mM dNTPs, 50 pmol of each primer, 1.25 U of Mango*Taq* polymerase (Bioline, London, UK) and DNA template - except for the negative (no-template) controls. Known test-positive, test-negative and no-template controls were included in every round of every PCR run. The cycling conditions for both primary and secondary (nested) PCRs were: 94 °C for 5 min (initial denaturation), followed by 35 cycles of 94 °C for 45 s (denaturation), 54 °C for 45 s (annealing) and 72 °C for 1 min (extension), followed by 72 °C for 10 min (final extension).

Amplicons were examined on ethidium bromide-stained 1.5% agarose gels using TBE (65 mM Tris-HCl, 27 mM boric acid, 1 mM EDTA, pH 9; Bio-Rad, Hercules, CA, USA) as the buffer, and their size estimated using a 100 bp-DNA ladder (Promega, Madison, WI, USA) as a reference. Amplicons were individually treated with ExoSAP-IT (Affymetrix, Santa Clara, CA, USA) according to the manufacturer’s instructions and directly sequenced (BigDye Terminator v.3.1 chemistry, Applied Biosystems, Foster City, CA, USA) using primers MSP-3 and MSP-4B in separate reactions. *ITS* sequences were aligned and examined using the program Geneious v.10 [24], and compared with other sequences acquired from the GenBank database (Additional file 2: Table S2). Sequence types or genotypes of *E. bieneusi* were named according to recent recommendations [1, 12]. *ITS* sequences were deposited in the GenBank database (NCBI) (accession nos. MG976812-MG976817).

### Nested PCR-based sequencing of *E. bieneusi SSU*

Selected genomic DNA samples that were test-positive for *ITS* were subjected to nested PCR-based sequencing of the small subunit of nuclear ribosomal DNA gene (*SSU*). In the first round of PCR, primers C1 (forward: 5'-CAC CAG GTT GAT TCT GCC-3') [25] and EBssuR2 (reverse: 5'-AAG CTC TTC ATC CCT ATG ACC ATC-3') (designed in the present study) were used to amplify 1216 bp of *SSU*. In the second round, primers EBIEF1 (forward: 5'-GAA ACT TGT CCA CTC CTT ACG-3') and EBIER1, 5'-CCA TGC ACC ACT CCT GCC ATT-3') [26] were employed to amplify a product of ~ 610 bp of *SSU*. PCR was conducted in 50 μl using the same buffer as employed for *ITS*. Known test-positive, test-negative and no-template controls were included in every round of every PCR run. For the first round, cycling conditions were: 94 °C for 5 min (initial denaturation), followed by 35 cycles of 94 °C for 1 min (denaturation), 56 °C for 1 min (annealing) and 72 °C for 1 min (extension), followed by 72 °C for 10 min (final extension). For the second round, cycling conditions were same except for denaturation (94 °C for 45 s) and annealing (64 °C for 45 s). Amplicons were examined, sized, purified and sequenced (same methods as for *ITS*) using primers EBIEF1 and EBIER1 in separate reactions. *SSU* sequences were examined and aligned using Geneious v.10 [24] and then compared with other sequences from the GenBank database (Additional file 3: Table S3). *SSU* sequences were deposited in the GenBank database (NCBI) (accession nos. MG976584-MG976586).

### Phylogenetic analysis of sequence data

*ITS* and *SSU* sequences from this and previous studies (cf. Additional file 2: Table S2 and Additional file 3: Table S3) were aligned separately over consensus lengths of 280 and 609 positions, respectively, and then subjected to phylogenetic analyses using the Bayesian inference (BI) and Monte Carlo Markov Chain (MCMC) methods in MrBayes v.3.2.3 [27]. The Akaike Information Criteria (AIC) test in jModeltest v.2.1.7 [28] was used to evaluate the likelihood parameters set for BI analysis. Posterior probability (pp) values were calculated by running 2,000,000 generations with four simultaneous tree-building chains, with trees saved every one hundredth generation. A 50% majority rule consensus tree for each analysis was constructed based on the final 75% of trees generated by BI. *Enterocytozoon bieneusi* clades and subclades were assigned using an established classification system [29, 30].

## Results

*Enterocytozoon* DNA was detected in 19 of 1365 (1.4%) faecal samples from wild marsupials, including eastern grey kangaroo (*n* = 13), swamp wallaby (*n* = 5) and common wombat (*n* = 1) from five of 11 water catchment areas by nested PCR of *ITS* (Additional file 1: Table S1). For all 11 catchments, the prevalences of *Enterocytozoon* were 2.0% (13/647) in the eastern grey kangaroo, 1.2% (5/403) in the swamp wallaby and 0.3% (1/315) in the common wombat. Within individual catchments, the highest prevalence (4.2%; 1/24) was recorded in catchment TAR, compared with 1.3–4.0% in the four other catchments (GV, MR, SV and YY). Test-positive samples were found in all seasons, with the highest prevalence (2.5%; 7/282) of *Enterocytozoon* in Spring; prevalences varied from 0–3.7% among years.

The sequencing of the 19 *ITS* amplicons (240–245 bp) and comparisons with reference sequences in the GenBank database identified five distinct sequence types (genotypes). One *E. bieneusi* genotype from the eastern grey kangaroo was NCF2 (matching a known sequence with accession no. KT750162) [31], and four were new genotypes (designated MWC_m1 to MWC_m4). Genotype MWC_m1 was from a common wombat; genotypes MWC_m2 and MWC_m3 were from swamp wallabies; and genotypes MWC_m2 and MWC_m4 were from eastern grey kangaroos. The mean G+C content (11.0%) of *ITS* sequences representing genotypes MWC_m2 to MWC_m4 was substantially less than those of the nine established Groups (1 to 9) of *E. bieneusi* genotypes (39.0–55.6%).

Genotype MWC_m1 from wombat differed by 1 bp (242/243; 99.6%) from the sequence with accession no. KT750162 (*E. bieneusi* genotype NCF2 from arctic fox) [31]. Genotypes MWC_m2, MWC_m3 and MWC_m4 each differed by 15 bp (229/244; 94.2%), 13 bp (232/245; 94.7%) and 2 bp (243/245; 99.2%) from the sequence with accession number KY706128 (i.e., representing *E. bieneusi* genotype CSK2 from red kangaroo (*Macropus rufus*); [11]). MWC_m4 was a “dominant” genotype in all three marsupial species studied here, having been detected in 6, 2 and 3 samples from catchment areas GV, SV and YY, respectively (Table 1).

**Table 1:** Genotypic designations for the operational taxonomic units (OTUs) of *Enterocytozoon* characterised by their internal transcribed spacer (*ITS*) of nuclear ribosomal DNA sequences (accession nos. listed) from 19 of 1365 individual faecal deposits (sample codes listed) from common wombats, eastern grey kangaroos and swamp wallabies, collected from five of Melbourne’s water catchment areas in different seasons and years

OTU/genotypic designation^a^	GenBank ID	Sample code	Host	Catchment area	Season	Year
NCF2	MG976814	GV6110	Kangaroo	Greenvale	Winter	2015
MWC_m1	MG976816	SV5961	Wombat	Silvan	Autumn	2015
MWC_m2	MG976812	SV4324	Kangaroo	Silvan	Summer	2014
MWC_m2	MG976815	MR3417	Wallaby	Maroondah	Autumn	2013
MWC_m2	MG976815	MR3433	Wallaby	Maroondah	Autumn	2013
MWC_m2	MG976815	TAR2172	Wallaby	Tarago	Winter	2011
MWC_m3	MG976817	MR7053	Wallaby	Maroondah	Winter	2016
MWC_m3	MG976817	MR7114	Wallaby	Maroondah	Winter	2016
MWC_m4	MG976813	GV4446	Kangaroo	Greenvale	Autumn	2014
MWC_m4	MG976813	GV4494	Kangaroo	Greenvale	Autumn	2014
MWC_m4	MG976813	GV4998	Kangaroo	Greenvale	Spring	2014
MWC_m4	MG976813	GV5007	Kangaroo	Greenvale	Spring	2014
MWC_m4	MG976813	GV5050	Kangaroo	Greenvale	Spring	2014
MWC_m4	MG976813	GV3007	Kangaroo	Greenvale	Spring	2012
MWC_m4	MG976813	SV3758	Kangaroo	Silvan	Winter	2013
MWC_m4	MG976813	SV4932	Kangaroo	Silvan	Winter	2014
MWC_m4	MG976813	YY3092	Kangaroo	Yan Yean	Spring	2012
MWC_m4	MG976813	YY3095	Kangaroo	Yan Yean	Spring	2012
MWC_m4	MG976813	YY3117	Kangaroo	Yan Yean	Spring	2012

^a^Genotypes MWC_m1 to MWC_m4 are all novel

**Fig. 1:**
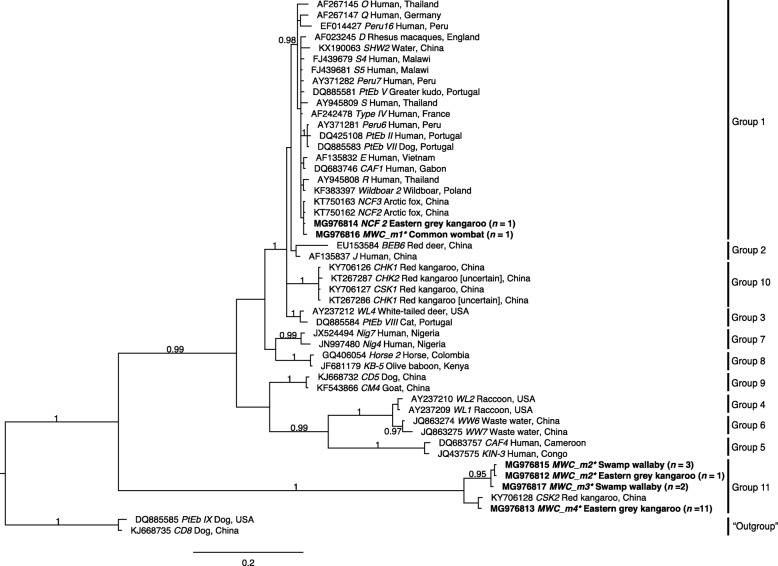
Phylogenetic analysis of internal transcribed spacer (*ITS*) of nuclear ribosomal DNA sequence data (Additional file 2: Table S2) by Bayesian inference (BI). Included here are *ITS* sequences of (i) *E. bieneusi* genotypes representing all currently recognised Groups (1 to 9) from the published literature; (ii) five genotypes of *Enterocytozoon* identified in the present study (bold-type); and (iii) the outgroup taxa. Groups 10 and 11 are proposed based on the results of the present analysis. Statistically significant posterior probabilities (pp) are indicated on branches. The scale-bar represents the number of substitutions per site

The *ITS* sequence data representing the five distinct genotypes (representing all 19 samples) were subjected to a phylogenetic analysis which included representative sequence data for 9 established groups of *E. bieneusi* (Fig. 1). The sequences of genotypes defined in this study were related to the currently recognised Groups (1 to 9) of *E. bieneusi*. Genotypes NCF2 and MWC_m1 could be assigned to Group 1, with strong statistical support (pp = 0.98). Genotypes CHK1 and CHK2 from an indeterminate species of macropod [10] as well as CSK1 from red kangaroo [11] in China all formed a new clade, namely Group 10, with strong statistical support (pp = 1.00). Genotypes MWC_m2 to MWC_m4 formed a new clade, namely Group 11 (pp = 1.00). Although Groups 1, 3, 5 to 11 were strongly supported (pp = 0.97 to 1.00), Groups 2 and 4 were not (pp = 0.55 to 0.90).

**Fig. 2:**
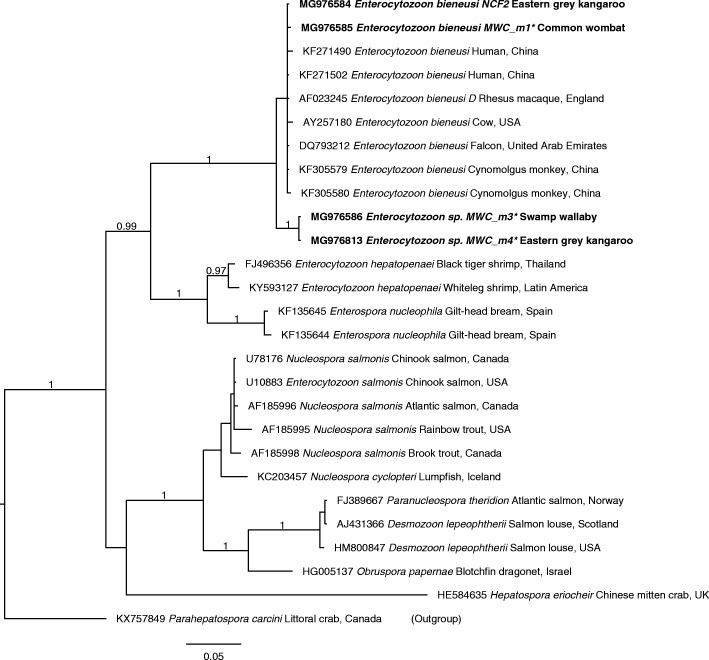
Phylogenetic analysis of small subunit of rDNA sequences (*SSU*) DNA sequence data (Additional file 3: Table S3) by Bayesian inference (BI). Included here are *SSU* sequences of (i) key taxa of the family Enterocytozoonidae, including representatives of *E. bieneusi* from the published literature; (ii) four operational taxonomic units (OTUs) or genotypes of *Enterocytozoon* identified in the present study (bold-type; cf. Fig. 1); and (iii) the outgroup: *Parahepatospora carcini*. Statistically significant posterior probabilities (pp) are indicated on branches. The scale-bar represents the number of substitutions per site

The relationship between novel genotypes MWC_m2 to MWC_m4 in this study and other species representing the family Enterocytozoonidae [32, 33] was inferred from a separate phylogenetic analysis of *SSU* sequence data using BI (Fig. 2; pairwise comparison of nucleotide differences given in Additional file 4: Table S4). The major clades were well-supported (pp = 0.97 to 1.00), most genotypes (MWC_m1, MWC_m3, MWC_m4 and NCF2; cf. Fig. 1) identified in this study represented the *E. bieneusi* clade. However, within this clade, there was limited support amongst most taxa. Representatives of *g*enotypes MWC_m3 and MWC_4 clustered together, and formed a separate clade (pp = 1.00) to the exclusion of all taxa representing *E. bieneusi* (Fig. 2).

## Discussion

This is the first molecular investigation of *Enterocytozoon* of marsupials in Australia. PCR-based sequencing of all 19 amplicons from 1365 faecal DNA samples (prevalence: 1.39%) revealed five operational taxonomic units (OTUs) of *Enterocytozoon* from a common wombat (*n* = 1), swamp wallabies (*n* = 5) and eastern grey kangaroos (*n* = 13) in Australia. In this study, we used oligonucleotide primers designed to flanking *SSU* and *LSU* regions of *E. bieneusi* to PCR-amplify rDNA products of 535–537 bp [23]. *ITS* sequence lengths obtained here were consistent with those (240–245 bp) for all nine recognised groups of *E. bieneusi* [29, 30], but distinct from other microsporidians, including *Encephalitozoon* spp. (*E. cuniculi*, *E. hellem* and *E. intestinalis*; 28–46 bp) [34], *Nucleospora cyclopteri* (261 bp) [35], *Nucleospora salmonis* (261 bp) [36] and *Obruspora papernae* (227 bp) [37].

When the 19 *ITS* amplicons derived from faecal DNAs from these marsupials were sequenced, and the five distinct sequences obtained were compared with those of *E. bieneusi* genotypes (*n* = 43) representing all nine recognised groups [29, 30], we identified genotype NCF2 from an eastern grey kangaroo and MWC_m1 from a common wombat. Phylogenetic analysis of *ITS* data showed that these two OTUs both clustered with genotypes recorded previously in humans, arctic fox, dog and wild boar [9] (Group 1; see Fig. 1). The relatedness of these OTUs with genotypes from humans (CAF1, D, E, Peru6, PtEb II, Q, S4, S5 and Type IV) might be an indication that they have zoonotic potential, but further study of many more samples from animals and humans is required to assess this proposal.

By contrast, the *ITS* sequences derived from the other three OTUs (MWC_m2 to MWC_m4 from eastern grey kangaroos or swamp wallabies) were highly divergent (57.4–64.4%) from those of any member of any of the ten recognised genotypic groups of *E. bieneusi* (Fig. 1)*.* Indeed, this divergence as well as AT-richness (89%) in *ITS* (but not in flanking *SSU* or *LSU* gene regions) did not allow a reliable alignment of homologous characters in the overall sequence alignment employed for phylogenetic analysis (Fig. 1), preventing a sound inference regarding the species or genotypic status of these three OTUs, particularly given their position relative to the outgroups (cf. Fig. 1).

Clearly, the sequences of the primers used in PCR were consistent with *E. bieneusi* to allow specific amplification, but sequence divergence in *ITS* prevented us from being able to reliably assign these three OTUs to any of the ten genotypic groups of *E. bieneusi*. Thus, we provisionally assigned them to a new group (Group 11) (Fig. 1). Given the substantial sequence divergence in *ITS*, we elected to use a more conserved rDNA gene region to investigate the taxonomic status of these four OTUs further. To do this, we designed new (forward and reverse) primers to *SSU* to PCR-amplify products of ~610 bp from faecal DNA samples in which we had identified representative, novel OTUs (MWC_m1, MWC_m3 and MWC_m4). Following the sequencing of these amplicons, we obtained *SSU* sequences which could be aligned (over a consensus length of 609 positions) with publicly available sequences (*n* = 23; 6 February 2018) representing seven genera (*Enterozytozoon*, *Enterospora*, *Hepatospora*, *Nucleospora*, *Obruspora*, *Parahepatospora* and *Paranucleospora*) and eight recognised species (including *E. bieneusi*, *E. hepatopenaei*, *E. salmonis* (syn. *Nucleospora salmonis*; [36]), *Enterospora nucleophila*, *Hepatospora eriocheir*, *Nucleospora cyclopteri*, *Obruspora papernae* and *Paranucleospora theridion* (syn. *Desmozoon lepeophtherii*; cf. [32]) and an outgroup, *Parahepatospora carcini*, within the family Enterocytozoonidae (Microsporidia) (Additional file 3: Table S3). In this case, homologous characters could be reliably aligned, and the phylogenetic analysis of the *SSU* data set showed that the novel OTUs (genotypes MWC_m3 and MWC_m4) grouped, with strong nodal support (pp = 1.00), with members of the genus *Enterocytozoon*, to the exclusion of all 16 other enterocytozoonid taxa included (Fig. 2). This analysis showed clearly that genotype MWC_m1 clustered with other members of the *E. bieneusi*-clade and thus represented *E. bieneusi*, and that MWC_m3 & MWC_m4 represented a new and distinct clade. As sequence variation (0–0.6%) representing the genetic sub-structuring within *E. bieneusi* was substantially less than differences (2.8–3.2%) between *E. bieneusi* genotypes and MWC_m3 & MWC_m4 upon pairwise comparison (Fig. 2), we propose that the latter two genotypes represent a novel species of *Enterocytozoon*. However, further work is required to sequence *SSU* from many more representatives of *Enterocytozoon*, in order to conduct a comprehensive phylogenetic analysis. The naming of a new species would need to follow all of the criteria set by the *International Code of Nomenclature for Algae, Fungi and Plants* [38].

There has been ongoing discussion about the species status of members of the genus *Enterocytozoon* [32, 36]. Currently, two species, namely *E. bieneusi* and *E. hepatopenaei*, are recognised [39, 40]. *Enterocytozoon bieneusi* is known to occur in eutherian mammals and in birds, and *E. hepatopenaei* has been recorded in crustaceans, such as the black tiger shrimp (*Penaeus monodon*) [40] and the whiteleg shrimp (*Penaeus vannamei*) [41]. However, due to limited *SSU* sequence similarity (85.2–85.9%) to *E. bieneusi* and a relatively close relationship with *Enterospora nucleophila* (cf. Fig. 2), there has been some reservation about the specific status of *E. hepatopenaei* (cf. [32]). With the present molecular findings, we now introduce added complexity by proposing that *Enterocytozoon* genotypes MWC_m2, MWC_m3 and MWC_m4 from the eastern grey kangaroo or swamp wallaby (cf. Figs. 1 and 2) represent a new species. Clearly, in the future, comprehensive phylogenetic analyses of nuclear genomic/ribosomal data sets (with suitable levels of phylogenetic signal) for a wide range of *Enterocytozoon* taxa should be undertaken to gain clarity as to the number of species within the genus. Provided sufficient amounts of genomic DNA of such taxa from broad host and distributional ranges can be acquired, a nuclear genomic sequencing-assembly effort, followed by phylogenetic analyses of tens, hundreds or thousands of homologous gene sequences (cf. [42]), could significantly assist toward solving the taxonomy/systematics of *Enterocytozoon.*

Interestingly, *E. bieneusi* genotypes from kangaroos from previous studies [10, 11] were assigned to (new) Groups 10 and 11. As these kangaroos had been kept in captivity in the Kangaroo Breeding Research Base, Jiangsu Province [11] and Zhengzhou Zoo, Henan Province [10] in China, it is unclear whether these *E. bieneusi* genotypes (CHK1, CHK2, CSK1 and CSK2) originated from Australia and were specifically affiliated to the hosts (indeterminate species) [10], or whether one or more of these genotypes might have been acquired from one or more other animal species in the same captive environment in China. Regardless, the distinctiveness of many *Enterocytozoon* genotypes from macropodid marsupials (Group 11, Fig. 1) and the AT-richness (88.6–89.4%) of the *ITS* sequences of these genotypes, with respect to those representing other groups, raises questions about the stringency of host specificity of these genotypes, and their co-evolution with their marsupial hosts. On a local scale, within a water catchment context, we now have the opportunity of evaluating, using established methods, whether any of the new *Enterocytozoon* genotypes (MWC_m1 to MWC_m4) recorded in marsupials also occur in other Australian-native and feral animals, or whether they are specific to macropods. On a national level, it would be interesting to extend molecular studies to a broader range of wild marsupial hosts in different states of Australia to gain a better understanding of the evolution of *Enterocytozoon*. Given that marsupials in Australia dispersed from South America 54.6–83.6 mya [43, 44], it would be interesting to explore, in an international context, whether, for example, kangaroos and wallabies in Australia harbour the same or similar *Enterocytozoon* taxa as their marsupial relatives in South America or other continents in a Gondwana context [44].

## Conclusions

Exploring the genetic composition of *Enterocytozoon bieneusi* in animals and humans is important for understanding transmission patterns of disease (enterocytozoonosis), and for its prevention and control. By conducting the present molecular-phylogenetic investigation of *Enterocytozoon* DNA in faecal deposits (*n* = 1365) from marsupials (common wombats, eastern grey kangaroos and swamp wallabies) living in catchment areas that supply the city of Melbourne (Australia) with drinking water, we identified or defined two operational taxonomic units (OTUs) or genotypes (NCF2 and MWC_m1) with zoonotic potential and three divergent genotypes (MWC_m2 to MWC_m4). Further studies of *Enterocytozoon* spp*.* from macropods and other animals should assist in elucidating the taxonomy and epidemiology of these species in Australia and elsewhere, and in assessing the public health risk of enterocytozoonosis.

## Additional files

Additional file 1: **Table S1.** The numbers of DNA samples from faecal deposits from common wombats (*Vombatus ursinus*), eastern grey kangaroos (*Macropus giganteus*) and swamp wallabies (*Wallabia bicolor*) collected from 11 of Melbourne’s water catchment areas from 2009 to 2017 and tested using PCR-based sequencing of the internal transcribed spacer (*ITS*) of nuclear ribosomal DNA. Within parentheses are the numbers of samples that were test-positive for *Enterocytozoon* using this technique and the codes representing currently recognised (NCF2) or novel (MWC_m1 to MWC_m4) operational taxonomic units (OTUs)/genotypes (cf. Table 1 and Additional file 2: Table S2). (XLSX 45 kb)

Additional file 2: **Table S2.** GenBank accession numbers of all internal transcribed spacer (*ITS*) of nuclear ribosomal DNA sequences used for phylogenetic analysis (Fig. 1), and associated information. Included here are *ITS* sequences of (i) *E. bieneusi* genotypes representing currently recognised Groups (1 to 9) from the published literature; (ii) five operational taxonomic units (OTUs)/genotypes of *Enterocytozoon* identified/defined in the present study; and (iii) the outgroups CD8 (KJ668735) and PtEbIX (DQ85585). (DOCX 75 kb)

Additional file 3: **Table S3.** GenBank accession numbers of all small subunit of ribosomal DNA (*SSU*) sequences used for phylogenetic analysis (Fig. 2), and associated information. Included here are *SSU* sequences of (i) key taxa of the family Enterocytozoonidae, including representatives of *E. bieneusi* from the published literature; (ii) four operational taxonomic units (OTUs)/genotypes of *Enterocytozoon* identified/defined in the present study; and (iii) the outgroup *Parahepatospora carcini. (DOCX 42 kb)*

Additional file 4: **Table S4.** Pairwise comparisons of differences/variation among the small subunit of nuclear ribosomal DNA gene sequences (*SSU*) (~609 bp consensus length) representing (i) four operational taxonomic units (OTUs)/genotypes of *Enterocytozoon* identified/defined in the present study (bold-type); (ii) eight species of of the family Enterocytozoonidae; (iii) and the outgroup *Parahepatospora carcini* used for phylogenetic analysis (cf. Fig. 2; Additional file 3: Table S3). (XLSX 54 kb)

## Abbreviations

AIC: Akaike information criteria
BI: Bayesian inference
*ITS*: Internal transcribed spacer of nuclear ribosomal DNA
*LSU*: Large subunit of nuclear ribosomal DNA
MCMC: Monte Carlo Markov Chain
OTU: Operational taxonomic unit
pp: Posterior probability
*SSU*: Small subunit of nuclear ribosomal DNA
