# Supplementary material for: New operational taxonomic units of Enterocytozoon in three marsupial species
Source: Parasit Vectors. 2018 Jun 28;11:371. doi: 10.1186/s13071-018-2954-x (PMC6022301; doi:10.1186/s13071-018-2954-x)
Supplement: Supplementary file 2 — Table S2. GenBank accession numbers of all internal transcribed spacer (ITS) of nuclear ribosomal DNA sequences used for phylogenetic analysis (Fig. 1), and associated information. Included here are ITS sequences of (i) E. bieneusi genotypes representing currently recognised Groups (1 to 9) from the published literature; (ii) five operational taxonomic units (OTUs)/genotypes of Enterocytozoon identified/defined in the present study; and (iii) the outgroups CD8 (KJ668735) and PtEbIX (DQ85585). (DOCX 75 kb) [file 13071_2018_2954_MOESM2_ESM.docx]

**Additional file 2: Table S2.** GenBank accession numbers of all internal transcribed spacer (*ITS*) of nuclear ribosomal DNA sequences used for phylogenetic analysis (Fig. 1), and associated information. Included here are *ITS* sequences of (i) *E. bieneusi* genotypes representing currently recognised Groups (1 to 9) from the published literature; (ii) five operational taxonomic units (OTUs)/genotypes of *Enterocytozoon* identified/defined in the present study; and (iii) the outgroups CD8 (KJ668735) and PtEbIX (DQ85585)

| GenBank ID | Genotype/  OTU | Origin | Country | Group | Reference |
| --- | --- | --- | --- | --- | --- |
| DQ683746 | CAF1 | *Homo sapiens* (human) | Gabon | 1 | [1] |
| AF023245 | D | *Macaca mulatta*  (rhesus macaque) | England | 1 | [2] |
| AF135832 | E | *Homo sapiens* (human) | Vietnam | 1 | [3] |
| KT750162 | NCF2 | *Vulpes lagopus*  (arctic fox) | China | 1 | [4] |
| KT750163 | NCF3 | *Vulpes lagopus*  (arctic fox) | China | 1 | [4] |
| AF267145 | O | *Homo sapiens* (human) | Thailand | 1 | [5] |
| EF014427 | Peru16 | *Homo sapiens* (human) | Peru | 1 | [6] |
| AY371281 | Peru6 | *Homo sapiens* (human) | Peru | 1 | [7] |
| AY371282 | Peru7 | *Homo sapiens* (human) | Peru | 1 | [7] |
| DQ425108 | PtEb II | *Homo sapiens* (human) | Portugal | 1 | [8] |
| DQ885581 | PtEb V | *Tragelaphus strepsiceros* (kudo) | Portugal | 1 | [9] |
| DQ885583 | PtEb VII | *Canis familiaris* (dog) | Portugal | 1 | [9] |
| AF267147 | Q | *Homo sapiens* (human) | Germany | 1 | [10] |
| AY945808 | R | *Homo sapiens* (human) | Thailand | 1 | [5] |
| AY945809 | S | *Homo sapiens* (human) | Thailand | 1 | [5] |
| FJ439679 | S4 | *Homo sapiens* (human) | Malawi | 1 | [11] |
| FJ439681 | S5 | *Homo sapiens* (human) | Malawi | 1 | [11] |
| KX190063 | SHW2 | Water | China | 1 | [12] |
| AF242478 | Type IV | *Homo sapiens* (human) | France | 1 | [13] |
| KF383397 | Wildboar2 | *Sus scrofa* (wild boar) | Poland | 1 | [14] |
| EU153584 | BEB6 | *Cervus elaphus*  (red deer) | China | 2 | [15] |
| AF135837 | J | *Homo sapiens* (human) | China | 2 | [16] |
| DQ885584 | PtEb VIII | *Felis catus* (cat) | Portugal | 3 | [9] |
| AY237212 | WL4 | *Odocoileus virginianus* (white-tailed deer) | USA | 3 | [17] |
| AY237209 | WL1 | *Procyon lotor* (raccoon) | USA | 4 | [18] |
| AY237210 | WL2 | *Procyon lotor* (raccoon) | USA | 4 | [18] |
| DQ683757 | CAF4 | *Homo sapiens* (human) | Cameroon | 5 | [1] |
| JQ437575 | KIN-3 | *Homo sapiens* (human) | Congo | 5 | [19] |
| JQ863274 | WW6 | Waste water | China | 6 | [20] |
| JQ863275 | WW7 | Waste water | China | 6 | [20] |
| JN997480 | Nig4 | *Homo sapiens* (human) | Nigeria | 7 | [21] |
| JX524494 | Nig7 | *Homo sapiens* (human) | Nigeria | 7 | [21] |
| GQ406054 | Horse 2 | *Equus ferus caballus* (horse) | Colombia | 8 | [22] |
| JF681179 | KB-5 | *Papio anubis*  (olive baboon) | Kenya | 8 | [23] |
| KJ668732 | CD5 | *Canis familiaris* (dog) | China | 9 | [24] |
| KF543866 | CM4 | *Capra aegagrus hircus* (goat) | China | 9 | [25] |
| KY706126 | CHK1 | *Macropus rufus*  (red kangaroo) | China | 10 | [26] |
| KT267286 | CHK1 | Indeterminate host species | China | 10 | [27] |
| KT267287 | CHK2 | Indeterminate host species | China | 10 | [27] |
| KY706127 | CSK1 | *Macropus rufus*  (red kangaroo) | China | 10 | [26] |
| KY706128 | CSK2 | *Macropus rufus*  (red kangaroo) | China | 10 | [26] |
| MG976814 | NCF2 | *Macropus giganteus* (eastern grey kangaroo) | Australia | 1 | This study |
| MG976816 | MWC_m1* | *Vombatus ursinus* (common wombat) | Australia | 1 | This study |
| MG976812 | MWC_m2* | *Macropus giganteus* (eastern grey kangaroo) | Australia | 11 | This study |
| MG976815 | MWC_m2* | *Wallabia bicolor*  (swamp wallaby) | Australia | 11 | This study |
| MG976817 | MWC_m3* | *Wallabia bicolor*  (swamp wallaby) | Australia | 11 | This study |
| MG976813 | MWC_m4* | *Macropus giganteus* (eastern grey kangaroo) | Australia | 11 | This study |
| KJ668735 | CD8 | *Canis familiaris* (dog) | China | Outgroup | [24] |
| DQ885585 | PtEb IX | *Canis familiaris* (dog) | USA | Outgroup | [28] |

* = a novel genotype identified in the present study

**References**

1. Breton J, Bart-Delabesse E, Biligui S, Carbone A, Seiller X, Okome-Nkoumou M, et al. New highly divergent rRNA sequence among biodiverse genotypes of *Enterocytozoon bieneusi* strains isolated from humans in Gabon and Cameroon. J Clin Microbiol. 2007;45(8):2580-9.

2. Chalifoux LV, Carville A, Pauley D, Thompson B, Lackner AA, Mansfield KG. *Enterocytozoon bieneusi* as a cause of proliferative serositis in simian immunodeficiency virus-infected immunodeficient macaques (*Macaca mulatta*). Arch Pathol Lab Med. 2000;124(10):1480-4.

3. Espern A, Morio F, Miegeville M, Illa H, Abdoulaye M, Meyssonnier V, et al. Molecular study of microsporidiosis due to *Enterocytozoon bieneusi* and *Encephalitozoon intestinalis* among human immunodeficiency virus-infected patients from two geographical areas: Niamey, Niger, and Hanoi, Vietnam. J Clin Microbiol. 2007;45(9):2999-3002.

4. Zhang XX, Cong W, Lou ZL, Ma JG, Zheng WB, Yao QX, et al. Prevalence, risk factors and multilocus genotyping of *Enterocytozoon bieneusi* in farmed foxes (*Vulpes lagopus*), northern China. Parasit Vectors. 2016;9(1):72.

5. Leelayoova S, Subrungruang I, Suputtamongkol Y, Worapong J, Petmitr PC, Mungthin M. Identification of genotypes of *Enterocytozoon bieneusi* from stool samples from human immunodeficiency virus-infected patients in Thailand. J Clin Microbiol. 2006;44(8):3001-4.

6. Cama VA, Pearson J, Cabrera L, Pacheco L, Gilman R, Meyer S, et al. Transmission of *Enterocytozoon bieneusi* between a child and guinea pigs. J Clin Microbiol. 2007;45(8):2708-10.

7. Sulaiman IM, Bern C, Gilman R, Cama V, Kawai V, Vargas D, et al. A molecular biologic study of *Enterocytozoon bieneusi* in HIV-infected patients in Lima, Peru. J Eukaryot Microbiol. 2003;50 Suppl 1:591-6.

8. Lobo ML, Xiao L, Antunes F, Matos O. Microsporidia as emerging pathogens and the implication for public health: a 10-year study on HIV-positive and -negative patients. Int J Parasitol. 2012;42(2):197-205.

9. Lobo ML, Xiao L, Cama V, Stevens T, Antunes F, Matos O. Genotypes of *Enterocytozoon bieneusi* in mammals in Portugal. J Eukaryot Microbiol. 2006;53:61-4.

10. Dengjel B, Zahler M, Hermanns W, Heinritzi K, Spillmann T, Thomschke A, et al. Zoonotic potential of *Enterocytozoon bieneusi*. J Clin Microbiol. 2001;39(12):4495-9.

11. ten Hove RJ, Van Lieshout L, Beadsworth MB, Perez MA, Spee K, Claas EC, et al. Characterization of genotypes of *Enterocytozoon bieneusi* in immunosuppressed and immunocompetent patient groups. J Eukaryot Microbiol. 2009;56(4):388-93.

12. Huang C, Hu Y, Wang L, Wang Y, Li N, Guo Y, et al. Environmental transport of emerging human-pathogenic *Cryptosporidium* species and subtypes through combined sewer overflow and wastewater. Appl Environ Microbiol. 2017;83(16):e00682-17.

13. Liguory O, Sarfati C, Derouin F, Molina JM. Evidence of different *Enterocytozoon bieneusi* genotypes in patients with and without human immunodeficiency virus infection. J Clin Microbiol. 2001;39(7):2672-4.

14. Němejc K, Sak B, Květoňová D, Hanzal V, Janiszewski P, Forejtek P, et al. Prevalence and diversity of *Encephalitozoon* spp. and *Enterocytozoon bieneusi* in wild boars (*Sus scrofa*) in Central Europe. Parasitol Res. 2014;113(2):761-7.

15. Zhao W, Zhang W, Wang R, Liu W, Liu A, Yang D, et al. *Enterocytozoon bieneusi* in sika deer (*Cervus nippon*) and red deer (*Cervus elaphus*): deer specificity and zoonotic potential of ITS genotypes. Parasitol Res. 2014;113(11):4243-50.

16. Zhang X, Wang Z, Su Y, Liang X, Sun X, Peng S, et al. Identification and genotyping of *Enterocytozoon bieneusi* in China. J Clin Microbiol. 2011;49(5):2006-8.

17. Guo Y, Alderisio KA, Yang W, Cama V, Feng Y, Xiao L. Host specificity and source of *Enterocytozoon bieneusi* genotypes in a drinking source watershed. Appl Environ Microbiol. 2014;80(1):218-25.

18. Sulaiman IM, Fayer R, Lal AA, Trout JM, Schaefer FW, Xiao L. Molecular characterization of microsporidia indicates that wild mammals harbor host-sdapted *Enterocytozoon* spp. as well as human-pathogenic *Enterocytozoon bieneusi*. Appl Environ Microbiol. 2003;69(8):4495-501.

19. Wumba R, Longo-Mbenza B, Menotti J, Mandina M, Kintoki F, Situakibanza NH, et al. Epidemiology, clinical, immune, and molecular profiles of microsporidiosis and cryptosporidiosis among HIV/AIDS patients. Int J Gen Med. 2012;5:603-11.

20. Li N, Xiao L, Wang L, Zhao S, Zhao X, Duan L, et al. Molecular surveillance of *Cryptosporidium* spp., *Giardia duodenalis*, and *Enterocytozoon bieneusi* by genotyping and subtyping parasites in wastewater. PLoS Negl Trop Dis. 2012;6(9):e1809.

21. Akinbo FO, Okaka CE, Omoregie R, Dearen T, Leon ET, Xiao L. Molecular epidemiologic characterization of *Enterocytozoon bieneusi* in HIV-infected persons in Benin City, Nigeria. Am J Trop Med Hyg. 2012;86(3):441-5.

22. Santín M, Vecino JAC, Fayer R. A zoonotic genotype of *Enterocytozoon bieneusi* in horses. J Parasitol. 2010;96(1):157-61.

23. Li W, Kiulia NM, Mwenda JM, Nyachieo A, Taylor MB, Zhang X, et al. *Cyclospora papionis*, *Cryptosporidium hominis,* and human-pathogenic *Enterocytozoon bieneusi* in captive baboons in Kenya. J Clin Microbiol. 2011;49(12):4326-9.

24. Karim MR, Dong H, Yu F, Jian F, Zhang L, Wang R, et al. Genetic diversity in *Enterocytozoon bieneusi* isolates from dogs and cats in China: host specificity and public health implications. J Clin Microbiol. 2014;52(9):3297-302.

25. Shi K, Li M, Wang X, Li J, Karim MR, Wang R, et al. Molecular survey of *Enterocytozoon bieneusi* in sheep and goats in China. Parasit Vectors. 2016;9(1):23.

26. Zhong Z, Tian Y, Song Y, Deng L, Li J, Ren Z, et al. Molecular characterization and multi-locus genotypes of *Enterocytozoon bieneusi* from captive red kangaroos (*Macropus rufus*) in Jiangsu province, China. PLoS One. 2017;12(8):e0183249.

27. Li J, Qi M, Chang Y, Wang R, Li T, Dong H, et al. Molecular characterization of *Cryptosporidium* spp., *Giardia duodenalis*, and *Enterocytozoon bieneusi* in captive wildlife at Zhengzhou Zoo, China. J Eukaryot Microbiol. 2015;62(6):833-9.

28. Feng Y, Li N, Dearen T, Lobo ML, Matos O, Cama V, et al. Development of a multilocus sequence typing tool for high-resolution genotyping of *Enterocytozoon bieneusi*. Appl Environ Microbiol. 2011;77(14):4822-8.
