# Supplementary material for: New operational taxonomic units of Enterocytozoon in three marsupial species
Source: Parasit Vectors. 2018 Jun 28;11:371. doi: 10.1186/s13071-018-2954-x (PMC6022301; doi:10.1186/s13071-018-2954-x)
Supplement: Supplementary file 3 — Table S3. GenBank accession numbers of all small subunit of ribosomal DNA (SSU) sequences used for phylogenetic analysis (Fig. 2), and associated information. Included here are SSU sequences of (i) key taxa of the family Enterocytozoonidae, including representatives of E. bieneusi from the published literature; (ii) four operational taxonomic units (OTUs)/genotypes of Enterocytozoon identified/defined in the present study; and (iii) the outgroup Parahepatospora carcini. (DOCX 42 kb) [file 13071_2018_2954_MOESM3_ESM.docx]

**Additional file 3: Table S3.** GenBank accession numbers of all small subunit of ribosomal DNA (*SSU*) sequences used for phylogenetic analysis (Fig. 2), and associated information. Included here are *SSU* sequences of (i) key taxa of the family Enterocytozoonidae, including representatives of *E. bieneusi* from the published literature; (ii) four operational taxonomic units (OTUs)/genotypes of *Enterocytozoon* identified/defined in the present study; and (iii) the outgroup *Parahepatospora carcini*

| GenBank ID | Taxon (genotype/OUT) | Host | Country | Reference |
| --- | --- | --- | --- | --- |
| AJ431366 | *Desmozoon lepeophtherii* | *Lepeophtheirus salmonis* (salmon louse) | Scotland | [1] |
| HM800847 | *Desmozoon lepeophtherii* | *Lepeophtheirus salmonis* (salmon louse) | USA | [2] |
| AY257180 | *Enterocytozoon bieneusi* | *Bos taurus* (cattle) | USA | [3] |
| KF305579 | *Enterocytozoon bieneusi* | *Macaca fascicularis* (cynomolgus monkey) | China | Unpublished |
| KF305580 | *Enterocytozoon bieneusi* | *Macaca fascicularis* (cynomolgus monkey) | China | Unpublished |
| DQ793212 | *Enterocytozoon bieneusi* | Falcon  (unknown species) | United Arab Emirates | [4] |
| KF271502 | *Enterocytozoon bieneusi* | *Homo sapiens* (human) | China | [5] |
| KF271490 | *Enterocytozoon bieneusi* | *Homo sapiens* (human) | China | [5] |
| AF023245 | *Enterocytozoon bieneusi* | *Macaca mulatta*  (rhesus macaques) | England | [6] |
| FJ496356 | *Enterocytozoon hepatopenaei* | *Penaeus monodon*  (black tiger shrimp) | Thailand | [7] |
| KY593127 | *Enterocytozoon hepatopenaei* | *Litopenaeus vannamei* (whiteleg shrimp) | Unknown | Unpublished |
| U10883 | *Enterocytozoon salmonis* | *Oncorhynchus tshawytscha*  (chinook salmon) | USA | [8] |
| KF135645 | *Enterospora nucleophila* | *Sparus aurata*  (gilt-head bream) | Spain | [9] |
| KF135644 | *Enterospora nucleophila* | *Sparus aurata*  (gilt-head bream) | Spain | [9] |
| HE584635 | *Hepatospora eriocheir* | *Eriocheir sinensis* (chinese mitten crab) | UK | [10] |
| KC203457 | *Nucleospora cyclopteri* | *Cyclopterus lumpus* (lumpfish) | The republic of Iceland | [11] |
| AF185996 | *Nucleospora salmonis* | *Salmo salar*  (atlantic salmon) | Canada | [12] |
| AF185998 | *Nucleospora salmonis* | *Salvelinus fontinalis* (brook trout) | Canada | [12] |
| U78176 | *Nucleospora salmonis* | *Oncorhynchus tshawytscha*  (chinook salmon) | Unknown | [13] |
| AF185995 | *Nucleospora salmonis* | *Oncorhynchus mykiss* (rainbow trout) | USA | [12] |
| HG005137 | *Obruspora papernae* | Blotchfin dragonet | Israel | [14] |
| FJ389667 | *Paranucleospora theridion* | *Salmo salar*  (atlantic salmon) | Norway | [15] |
| MG976584 | *Enterocytozoon bieneusi* (NCF2) | *Macropus giganteus* (eastern grey kangaroo) | Australia | This study |
| MG976585 | *Enterocytozoon bieneusi* (MWC_m1*) | *Vombatus ursinus* (common wombat) | Australia | This study |
| MG976586 | *Enterocytozoon* sp. (MWC_m3*) | *Wallabia bicolor*  (swamp wallaby) | Australia | This study |
| MG976813 | *Enterocytozoon* sp. (MWC_m4*) | *Macropus giganteus* (eastern grey kangaroo) | Australia | This study |
| KX757849 | *Parahepatospora carcini* | *Carcinus maenas*  (littoral crab) | Canada | [16] |

*Enterocytozoon salmonis* and *Nucleospora salmonis* are synonyms. *Desmozoon lepeophtherii* and *Paranucleospora theridion* are synonyms. Genotypes of *Enterocytozoon* identified in the present study based on the internal transcribed spacer (*ITS*) of nuclear ribosomal DNA sequence are shown in parentheses. * = a novel genotype

**References**

1. Freeman MA, Sommerville C. *Desmozoon lepeophtherii* n. gen., n. sp., (Microsporidia: Enterocytozoonidae) infecting the salmon louse *Lepeophtheirus salmonis* (Copepoda: Caligidae). Parasit Vectors. 2009;2(1):58.

2. Jones SR, Prosperi‐Porta G, Kim E. The diversity of microsporidia in parasitic copepods (Caligidae: Siphonostomatoida) in the northeast pacific ocean with description of *Facilispora margolisi* ng, n. sp. and a new family Facilisporidae n. fam. J Eukaryot Microbiol. 2012;59(3):206-17.

3. Fayer R, Santín M, Trout J. First detection of microsporidia in dairy calves in north America. Parasitol Res. 2003;90(5):383-6.

4. Müller MG, Kinne J, Schuster RK, Walochnik J. Outbreak of microsporidiosis caused by *Enterocytozoon bieneusi* in falcons. Vet Parasitol. 2008;152(1):67-78.

5. Liu H, Shen Y, Yin J, Yuan Z, Jiang Y, Xu Y, et al. Prevalence and genetic characterization of *Cryptosporidium*, *Enterocytozoon*, *Giardia* and *Cyclospora* in diarrheal outpatients in China. BMC Infect Dis. 2014;14:25.

6. Chalifoux LV, Carville, A., Pauley, D., Thompson, B., Lackner, A.A., Mansfield KG. *Enterocytozoon bieneusi* as a cause of proliferative serositis in simian immunodeficiency virus–infected immunodeficient macaques (*Macaca mulatta*). Arch Pathol Lab Med. 2000;124(10):1480-4.

7. Tourtip S, Wongtripop S, Stentiford GD, Bateman KS, Sriurairatana S, Chavadej J, et al. *Enterocytozoon hepatopenaei* sp. nov. (Microsporida: Enterocytozoonidae), a parasite of the black tiger shrimp *Penaeus monodon* (Decapoda: Penaeidae): fine structure and phylogenetic relationships. J Invertebr Pathol. 2009;102(1):21-9.

8. Barlough J, McDowell T, Milani A, Bigornia L, Slemenda S, Pieniazek N, et al. Nested polymerase chain reaction for detection of *Enterocytozoon salmonis* genomic DNA in chinook salmon *Oncorhynchus tshawytscha*. Dis Aquat Organ. 1995;23(1):17-23.

9. Palenzuela O, Redondo MJ, Cali A, Takvorian PM, Alonso-Naveiro M, Alvarez-Pellitero P, et al. A new intranuclear microsporidian, *Enterospora nucleophila* n. sp., causing an emaciative syndrome in a piscine host (*Sparus aurata*), prompts the redescription of the family Enterocytozoonidae. Int J Parasitol. 2014;44(3):189-203.

10. Stentiford G, Bateman K, Dubuffet A, Chambers E, Stone D. *Hepatospora eriocheir* (Wang and Chen, 2007) gen. et comb. nov. infecting invasive Chinese mitten crabs (*Eriocheir sinensis*) in Europe. J Invertebr Pathol. 2011;108(3):156-66.

11. Freeman MA, Kasper JM, Kristmundsson Á. *Nucleospora cyclopteri* n. sp., an intranuclear microsporidian infecting wild lumpfish, *Cyclopterus lumpus* L., in icelandic waters. Parasit Vectors. 2013;6:49.

12. Gresoviac SJ, Khattra JS, Nadler SA, Kent ML, Devlin RH, Vivares CP, et al. Comparison of small subunit ribosomal RNA gene and internal transcribed spacer sequences among isolates of the intranuclear microsporidian *Nucleospora salmonis*. J Eukaryot Microbiol. 2000;47(4):379-87.

13. Docker MF, Kent ML, Hervio DM, Khattra JS, Weiss LM, Cali A, et al. Ribosomal DNA sequence of *Nucleospora salmonis* Hedrick, Groff and Baxa, 1991 (Microsporea: Enterocytozoonidae): implications for phylogeny and nomenclature. J Eukaryot Microbiol. 1997;44(1):55-60.

14. Diamant A, Rothman SB, Goren M, Galil BS, Yokes MB, Szitenberg A, et al. Biology of a new xenoma-forming gonadotropic microsporidian in the invasive blotchfin dragonet *Callionymus filamentosus*. Dis Aquat Organ. 2014;109(1):35-54.

15. Nylund S, Nylund A, Watanabe K, Arnesen CE, Karlsbakk E. *Paranucleospora theridion* n. gen., n. sp. (Microsporidia, Enterocytozoonidae) with a life cycle in the Salmon louse (*Lepeophtheirus salmonis,* Copepoda) and Atlantic salmon (*Salmo salar*). J Eukaryot Microbiol. 2010;57(2):95-114.

16. Bojko J, Clark F, Bass D, Dunn AM, Stewart-Clark S, Stebbing PD, et al. *Parahepatospora carcini* n. gen., n. sp., a parasite of invasive *Carcinus maenas* with intermediate features of sporogony between the *Enterocytozoon* clade and other microsporidia. J Invertebr Pathol. 2017;143:124-34.
